# Sweat Electrolyte Profiles During Daily Physical Activities Among Chinese Adults

**DOI:** 10.3390/nu18101531

**Published:** 2026-05-12

**Authors:** Yan Chen, Yiheng Liang, Zhihui Lu, Zhirui Zhang, Wei Wen, Chengnan Zhang, Feng Liu, Mo Wang, Meiyuan Feng, Junqiang Qiu

**Affiliations:** 1 Department of Exercise Biochemistry, Exercise Science School, Beijing Sport University, Beijing 100084, China; ryannchen@bsu.edu.cn (Y.C.); liamyiheng5373@sina.com (Y.L.); 2022210559@bsu.edu.cn (Z.L.); zzrdanb@163.com (Z.Z.); 2024110069@bsu.edu.cn (W.W.); 2023992990@bsu.edu.cn (C.Z.); 2 PepsiCo Asia R&D Center, Shanghai 201114, China; feng.liu@pepsico.com (F.L.); tracy.m.wang@pepsico.com (M.W.); 3 Beijing Sports Nutrition Engineering Research Center, Beijing 100084, China; 4 School of Public Health, Southeast University, Nanjing 210018, China

**Keywords:** sweat rate, electrolyte composition, hydration, Chinese adults, sex differences

## Abstract

**Background:** Recreational exercise has become increasingly common among Chinese adults. However, population-specific data on sweat rate and electrolyte composition during typical daily physical activities remain limited. Therefore, this study aimed to comprehensively characterize the hydration status, sweat rate, and sweat electrolyte composition among Chinese adults engaging in common daily exercises under controlled environmental conditions, and to examine sex-related differences. **Methods:** In this cross-sectional study, 285 healthy adults (143 men, 142 women) were assigned to one of three separate activity groups: brisk walking (n = 100), running (n = 90), or cycling (n = 95). Activity protocols were standardized using fixed activity-specific speeds corresponding to 5.2, 8.2, and 4.4 METs for brisk walking, running, and cycling, respectively, based on the Chinese Compilation of Physical Activities. Each participant completed one 60-min exercise session under controlled environmental conditions. Sweat samples were collected from the chest using sweat patches and analyzed for Na^+^, K^+^, Ca^2+^, Mg^2+^, Fe, Zn^2+^, and Cu^2+^ using ICP-MS/OES. Whole-body sweat sodium and potassium concentrations were estimated using validated regression equations. Body mass loss (BML) and sweat rate were calculated from pre- and post-exercise nude body mass. **Results**: Across all participants and activity types, the overall mean sweat rate was 0.71 ± 0.28 L/h, and the mean percentage of body mass loss (BML%) was 0.78 ± 0.45%. Among the three physical activities, running elicited higher sweat rates (0.92 ± 0.29 L/h) and BML% (1.16 ± 0.36%) than brisk walking or cycling (*p* < 0.05). The estimated whole-body Na^+^ and K^+^ concentrations across all participants were 34.10 ± 10.31 mmol/L and 3.35 (3.02–3.93) mmol/L, respectively, with 59.3% of participants classified as having moderate Na^+^ levels (30–60 mmol/L). Men exhibited higher sweat rates and Na^+^ concentrations, whereas women showed higher K^+^, Zn^2+^, and Cu^2+^ levels (*p* < 0.05). **Conclusions**: Chinese adults engaging in common daily physical activities under temperate environmental conditions demonstrated low-to-moderate sweat rates and sodium concentrations. These findings provide baseline reference data for population-specific hydration education and may inform future validation and application of wearable sweat-sensing technologies in public health monitoring.

## 1. Introduction

In recent years, recreational physical activity has become an integral part of daily life for Chinese adults. According to national fitness surveys, more than 600 million adults in China engage in regular exercise at least once per week, with brisk walking, running, and cycling being the most popular activities, typically lasting around one hour per session. During physical activities, the body dissipates heat primarily through sweating, leading to fluid and electrolyte losses [1,2]. If these losses are not adequately replaced, disturbances in water and electrolyte balance may occur, resulting in dehydration, impaired thermoregulation, and reduced physical performance [3]. Consequently, understanding sweat and hydration characteristics during common daily physical activities is crucial for promoting safe exercise participation and for developing appropriate, population-specific hydration strategies to support the rapidly growing fitness community in China. During physical activity, fluid and electrolyte losses through sweating are closely related to individual sweating rate and sweat electrolyte concentration [3,4,5]. These parameters vary widely among individuals and populations, influenced by genetic factors, lifestyle, exercise type and intensity, and environmental conditions [3].

Most previous studies on sweat rate and electrolyte composition have focused on athletes or high-intensity exercise performed under hot or humid environments [6,7,8,9,10,11,12]. These investigations have established normative data for sweat sodium and potassium concentrations and have clarified physiological mechanisms underlying sweat secretion and electrolyte reabsorption. However, the findings from trained Western populations may not accurately represent the sweat responses of the general Chinese population engaged in moderate-intensity daily activities. Chinese adults may differ from those reported in Western populations in several physiological and environmental aspects that can influence sweating behavior and electrolyte balance. Habitual sodium intake in China is reported to exceed 6000 mg/day, and the average daily salt consumption in 2020 was 9.3 g—still higher than the national dietary recommendation of 5 g/day [13,14]. Chronic high sodium intake may alter body fluid distribution and renal sodium handling, potentially affecting sweat gland reabsorption capacity and overall hydration status [15,16]. In addition, average body size and total body water content are lower than those of Western adults, which may further modify thermoregulatory and sweating responses [17]. These population-specific characteristics underscore the need for localized investigations of sweat rate and electrolyte composition to provide population-specific reference data for future hydration education and research among Chinese adults.

Despite the rapid growth of fitness participation nationwide, systematic data on sweat rate, body mass loss, and electrolyte concentrations during typical daily physical activities remain scarce. This knowledge gap limits the development of population-specific reference values and evidence-based hydration education for daily physical activity settings. Therefore, the present study was designed to characterize sweat rate, body mass loss, and sweat electrolyte profiles among healthy Chinese adults during three common daily physical activities—brisk walking, running, and cycling—and to compare these responses across activity modes and between sexes. We hypothesized that sweat rate, body mass loss, and sweat electrolyte profiles would differ across activity modes and between sexes. The findings are expected to provide baseline data for understanding sweat and hydration characteristics in the Chinese population and to inform future hydration research, public health-oriented hydration education, and the design of intervention studies for daily exercise settings.

## 2. Materials and Methods

### 2.1. Participants

This study aimed to investigate sweat characteristics in Chinese adults during daily physical activities, including three common exercise modes: brisk walking, running, and cycling. Participants were recruited through online and community advertisements. Eligibility criteria were as follows: (1) age between 18 and 60 years; (2) engagement in low-to-moderate intensity exercise for at least 30 min on ≥3 days per week; (3) apparently healthy, with no clinically diagnosed diseases; (4) non-smokers and non-drinkers with no other unhealthy lifestyle habits; (5) ability and willingness to comply with all experimental procedures; (6) provision of written informed consent after being informed of study risks and benefits; and (7) no participation in any other clinical or dietary intervention study within the previous month. All participants completed a standardized health screening questionnaire before enrollment. The study protocol was reviewed and approved by the Sports Science Experiment Ethics Committee of Beijing Sport University (No. 2024454H) and conducted in accordance with the Declaration of Helsinki.

Sample size was calculated using G*Power software (version 3.1.9.7), assuming an effect size of 0.20, significance level (α) of 0.05, and statistical power (1 − β) of 0.80. The required minimum sample size was 82 participants per group. A total of 285 adults were finally included in the study, comprising 143 men and 142 women.

### 2.2. Study Design and Procedures

This cross-sectional study was conducted in Beijing, China, in December 2024. All experimental sessions were performed under controlled environmental conditions (ambient temperature 23.0 ± 1.3 °C, relative humidity 23.6 ± 2.9%). The experiment consisted of a baseline assessment phase and an exercise testing session. During the baseline assessment, participants completed a questionnaire on physical activity level and underwent measurements of height, body mass, and body composition. Following baseline testing, participants were assigned to one of three separate activity groups—brisk walking, running, or cycling—each representing a typical mode of daily physical activity among Chinese adults. Each participant completed only one 60-min exercise session in the assigned activity group under the same controlled environmental conditions. To reduce the potential influence of large differences in dietary intake, participants were provided with a standardized meal plan for the day before the experimental trial and were instructed to follow this plan as closely as possible. On the morning of the experimental day, all participants were provided with a standardized breakfast before testing. To standardize pre-exercise hydration status and reduce the influence of diurnal variation, all experimental trials were conducted in the morning. To ensure adequate hydration prior to exercise, participants were instructed to consume 500 mL of commercially bottled purified water 2 h before testing. Urine samples were collected the day before, the morning of, and immediately before the exercise trial, and only those with a urine specific gravity (USG) ≤ 1.020 were allowed to start the exercise session. During exercise, participants were allowed to drink commercially bottled purified water freely, and their fluid intake was precisely recorded. Nude body mass was measured immediately before and after exercise to determine sweat loss, and sweat samples were collected throughout the activity session for subsequent electrolyte analysis.

### 2.3. Exercise Protocol

To simulate common daily physical activities among Chinese adults, three exercise modalities were selected: brisk walking, running, and cycling. Activity protocols were standardized within each exercise mode using fixed activity-specific speeds and corresponding metabolic equivalent (MET) values derived from the Chinese Compilation of Physical Activities in Healthy Adults Aged 18–64 (CCPA) [18], rather than %VO_2_max, %HRmax, or cycling power. The CCPA is a Chinese population-specific reference standard for estimating physical activity energy expenditure and was developed based on published literature and laboratory measurements, including indirect calorimetry measurements for selected physical activities. According to the CCPA, the target speeds were set at 6 km/h for brisk walking, 8 km/h for running, and 13 km/h for cycling, corresponding to 5.2, 8.2, and 4.4 METs, respectively. These protocols were selected to represent common daily physical activity scenarios among Chinese adults and to characterize activity-specific exercise load using a Chinese population-based reference. Each participant completed 60 min of continuous exercise in the assigned activity group. Heart rate was continuously recorded using a heart rate monitor (Polar Verity Sense, Sport Konsulting, Krakow, Poland), and ratings of perceived exertion (RPE) were recorded at 0, 15, 30, and 45 min and immediately post-exercise using the Borg 6–20 scale to characterize the actual exercise load.

### 2.4. Sweat Collection and Analysis

Local sweat samples were collected from the chest region using absorbent sweat patches (Tegaderm+ Pad, 3M, St. Paul, MN, USA) [6,19]. Before exercise, the chest skin surface was carefully cleaned with alcohol and deionized water and then dried with sterile wipes (Wypall L-40, Kimberly-Clark, Irving, TX, USA) to minimize contamination from skin surface residues. Sweat patches were applied immediately after skin preparation and remained in place throughout the exercise session. After exercise, the absorbent pad was removed using sterile tweezers, and sweat was extracted using a disposable syringe. Extracted sweat samples were transferred into sterile microtubes, sealed, and immediately stored at −20 °C until analysis to prevent evaporation and contamination.

Electrolyte concentrations were determined using inductively coupled plasma mass spectrometry (ICP-MS; iCAP RQ, Thermo Scientific, Waltham, MA, USA) for Na^+^, K^+^, Ca^2+^, and Mg^2+^, and inductively coupled plasma optical emission spectrometry (ICP-OES; TOPEX, Perkin Elmer, Waltham, MA, USA) for Fe (Fe^2+^ + Fe^3+^), Zn^2+^, and Cu^2+^. Although these procedures were used to minimize contamination, no analyte-specific patch blank correction was applied for Ca^2+^, Mg^2+^, Fe, Zn^2+^, or Cu^2+^. Therefore, these analytes were reported as local chest sweat mineral concentrations.

### 2.5. Hydration Assessment

Nude body mass was measured immediately before and after exercise using an electronic scale (Yingheng Electronic Technology Co., Ltd., Dongguan, China) with an accuracy of 0.01 kg. Participants were instructed to void before the pre-exercise measurement, remove all clothing, jewelry, and accessories, and ensure that the skin was completely dry before each weighing.

Percentage body mass loss (BML%) was calculated from the net change in nude body mass before and after exercise as follows:

BML% = [(BW_pre_ − BW_post_)/BW_pre_] × 100%

Sweat rate (L/h) was calculated according to the method proposed by Baker et al. [6] as follows:

$$\mathrm{Sweat}\;\mathrm{rate}\;(L/h)=\frac{{\mathrm{BW}}_{p\mathrm{re}}-({\mathrm{BW}}_{p\mathrm{ost}}\;-\;\mathrm{FI}\;+\;\mathrm{UO})}{\mathrm{Exercise}\;\mathrm{time}\;(h)}$$

BW_pre_ and BW_post_ represent pre- and post-exercise nude body mass (kg), and FI is the amount of fluid ingested during exercise (kg), and UO represents urine output during the exercise session (kg). No urine output occurred during the 60-min exercise sessions; therefore, UO was recorded as zero in the calculation.

### 2.6. Sweat Electrolyte Calculation and Whole-Body Estimation

Sweat electrolyte concentrations obtained from raw measurements were converted using the following equations:

$${{\mathrm{Na}}^{+}}_{\mathrm{chest}}\;\left(\mathrm{mmol}/L\right)\;=\;\frac{{{\mathrm{Na}}^{+}}_{\mathrm{raw}\;}\left(\mathrm{mg}/\mathrm{kg}\right)}{23}$$

$${K^{+}}_{\mathrm{chest}}\;\left(\mathrm{mmol}/L\right)=\frac{{K^{+}}_{\mathrm{raw}\;}\left(\mathrm{mg}/\mathrm{kg}\right)}{39}$$

$${{\mathrm{Ca}}^{2+},\;{\mathrm{Mg}}^{2+},\;\mathrm{Fe},\;{\mathrm{Zn}}^{2+},\;{\mathrm{Cu}}^{2+}}_{\mathrm{chest}}\;\left(\mathrm{mg}/L\right)=\mathrm{raw}\;\mathrm{concentration}\;(\mathrm{mg}/\mathrm{kg})$$

Whole-body sweat sodium and potassium concentrations were estimated based on established regression equations proposed by Baker et al. [7,19]. The background sodium concentration of the patch (Na^+^_background_) was calculated as:

Na^+^_background_ (mmol/L) = −4.377 × ln (sweat collected (g)) + 4.3

The whole-body sodium (Na^+^_whole_) and potassium (K^+^_whole_) concentrations were then calculated as:

Na^+^_whole_ (mmol/L) = 0.51 × [Na^+^_chest_ − Na^+^_background_] + 6.73

For potassium, no patch background correction was applied. Estimated whole-body potassium concentration (K^+^_whole_) was calculated directly from chest sweat potassium using the following equation:

K^+^_whole_ (mmol/L) = 0.76 × K^+^_chest_ + 0.55

These regression equations were applied only to Na^+^ and K^+^. These equations were originally validated in physically active adult participants under controlled exercise or exercise-heat stress conditions. Therefore, in the present study, Na^+^_whole_ and K^+^_whole_ are reported as estimated whole-body sweat electrolyte concentrations rather than directly measured whole-body values. For Ca^2+^, Mg^2+^, Fe, Zn^2+^, and Cu^2+^, no validated site-specific correction or whole-body estimation equations were available; therefore, these analytes were reported as local chest sweat concentrations rather than estimated whole-body concentrations.

According to Lara et al. [20], whole-body sweat sodium concentration (Na^+^_whole_) was classified as low (<30 mmol/L), moderate (30–60 mmol/L), or high (>60 mmol/L). Frequency distributions were plotted to describe the variability in sweat sodium concentrations among participants.

### 2.7. Statistical Analysis

All statistical analyses were performed using IBM SPSS Statistics (version 27.0.1.0, IBM Corp., Armonk, NY, USA). The Shapiro–Wilk test was used to assess data normality, and Levene’s test was used to evaluate homogeneity of variance before parametric comparisons. Normally distributed data are expressed as mean ± standard deviation (SD), whereas non-normally distributed data are presented as median (interquartile range), M (IQR).

For normally distributed variables with homogeneous variances, differences among exercise modes were analyzed using one-way analysis of variance (ANOVA), followed by Bonferroni-adjusted post hoc tests when significant main effects were detected. When the assumption of homogeneity of variance was not met, Welch’s ANOVA was applied, followed by Games–Howell post hoc tests. For non-normally distributed variables, comparisons among exercise modes were performed using the Kruskal–Wallis test, followed by Bonferroni-adjusted pairwise comparisons when appropriate. For sex-related comparisons, two-way ANOVA was used with sex and activity mode as fixed factors. For sweat rate and estimated whole-body sweat Na^+^ concentration, two-way ANCOVA was applied with body mass as the covariate. When sex-specific comparisons within each activity group were reported, independent-samples *t*-tests or Mann–Whitney U tests were used according to data distribution. Statistical significance was set at *p* < 0.05.

## 3. Results

### 3.1. Participant Characteristics

A total of 285 adults (143 male and 142 female) completed the study. Participants were assigned to three separate activity groups: brisk walking (n = 100; 49 male and 51 female), running (n = 90; 43 male and 47 female), and cycling (n = 95; 51 male and 44 female).

Table 1 summarizes the anthropometric and body composition characteristics of the participants. No significant differences were observed among the three activity groups in age, height, body mass, body mass index (BMI), body surface area (BSA), muscle mass, or body fat percentage (all *p* > 0.05).

### 3.2. Sweat Rate and Body Mass Loss

**Figure 1:**
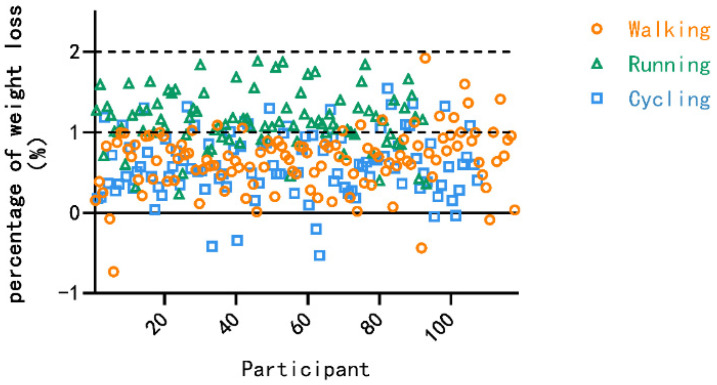
Distribution of BML% during different physical activities among Chinese adults.

Body mass decreased significantly after exercise across all activity types. The body mass loss percentage (BML%) and sweat rate of Chinese adults during daily physical activities are summarized in Table 2. The mean BML% differed significantly among activity types (F = 61.883, *p* < 0.001, partial η^2^ = 0.305). Running resulted in greater BML% than brisk walking (mean difference = 0.406%, 95% CI: 0.305 to 0.506, *p* < 0.001) and cycling (mean difference = 0.410%, 95% CI: 0.308 to 0.512, *p* < 0.001), while brisk walking and cycling were not significantly different. The distribution of BML% across different physical activities among Chinese adults is presented in Figure 1, showing that most individuals experienced a reduction of less than 2% of pre-exercise body mass, with no participants exceeding this threshold. These between-activity differences should be interpreted in relation to the activity-specific MET values, as the running protocol had a higher estimated metabolic cost than the brisk walking and cycling protocols.

**Figure 2:**
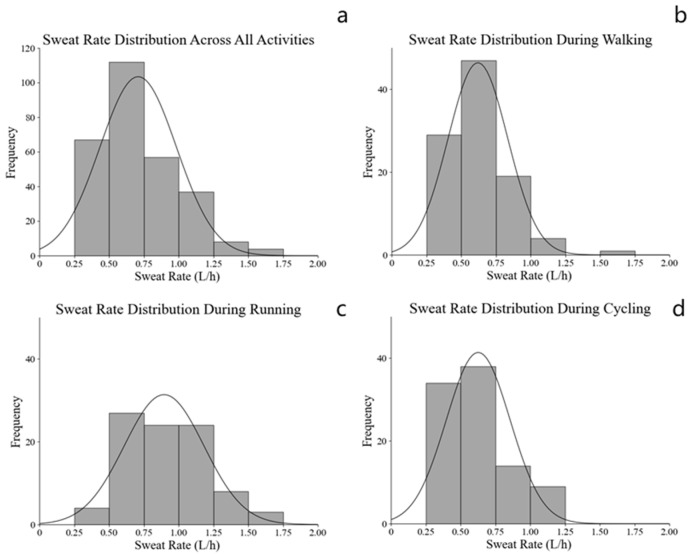
Frequency distribution of sweat rate across all activities (**a**) and by activity type: (**b**) brisk walking, (**c**) running, and (**d**) cycling.

The overall sweat rate across all participants was 0.71 ± 0.28 L/h. Sweat rate differed significantly among activity modes, with a large effect size (F = 37.876, *p* < 0.001, partial η^2^ = 0.212). Post hoc comparisons showed that running elicited a higher sweat rate than brisk walking (mean difference = 0.275 L/h, 95% CI: 0.189 to 0.361, *p* < 0.001) and cycling (mean difference = 0.269 L/h, 95% CI: 0.182 to 0.356, *p* < 0.001), whereas brisk walking and cycling did not differ significantly (*p* > 0.05). The overall distribution of sweat rates exhibited a slightly right-skewed pattern, with most values clustering between 0.5 and 0.75 L/h, as shown in Figure 2. A consistent pattern was observed across the brisk walking, running, and cycling groups, where sweat rates were predominantly concentrated within the 0.5–0.75 L/h range.

When stratified by sex, male participants had higher sweat rates than female participants in all three activity types (*p* < 0.05). Within-sex comparisons showed that running elicited greater sweat rates than both brisk walking and cycling in men and in women (*p* < 0.05), whereas brisk walking and cycling did not differ within the same sex (*p* > 0.05) (Table 3).

### 3.3. Sweat Electrolyte Concentrations

#### 3.3.1. Local Sweat Electrolyte Concentrations

The concentrations of sweat electrolytes measured from the chest region during daily physical activities are presented in Table 4. Among all electrolytes measured in chest sweat, Na^+^ and K^+^ showed the highest concentrations, whereas Ca^2+^, Mg^2+^, Fe, Zn^2+^, and Cu^2+^ were relatively lower. There were no significant differences in Na^+^_chest_ and K^+^_chest_ concentrations among the brisk walking, running, and cycling groups (*p* > 0.05). For the trace minerals, the Zn^2+^_chest_ concentration in the running group was significantly lower than in the walking and cycling groups (*p* < 0.05), while no significant differences were observed in Ca^2+^_chest_, Mg^2+^_chest_, Fe_chest_, or Cu^2+^_chest_ concentrations among activities.

Sex-specific comparisons of local sweat electrolyte concentrations are shown in Table 5. Across all activity types, men exhibited significantly higher Na^+^_chest_ concentrations than women (*p* < 0.05), whereas female participants showed higher K^+^_chest_, Zn^2+^_chest_, and Cu^2+^_chest_ concentrations (*p* < 0.05). No significant sex differences were observed in Ca^2+^_chest_, Mg^2+^_chest_, or Fe_chest_ concentrations (*p* > 0.05).

Within each activity, male participants in the brisk walking group had higher Na^+^_chest_ concentrations than women, while K^+^_chest_, Mg^2+^_chest_, Fe_chest_, and Cu^2+^_chest_ were significantly lower in males (*p* < 0.05). In the running group, male participants had higher Na^+^_chest_ concentrations but lower K^+^_chest_, Zn^2+^_chest_, and Cu^2+^_chest_ concentrations compared with females (*p* < 0.05). In the cycling group, Na^+^_chest_ concentrations did not differ significantly between genders, but female participants showed higher K^+^_chest_, Zn^2+^_chest_, and Cu^2+^_chest_ concentrations (*p* < 0.05).

Across different activity types, male runners exhibited significantly lower Ca^2+^_chest_ and Zn^2+^_chest_ concentrations than male walkers and cyclists (*p* < 0.05), while no significant differences were observed between the walking and cycling groups. Similarly, female runners had lower Zn^2+^_chest_ concentrations than female walkers and cyclists (*p* < 0.05), with no significant difference between the walking and cycling groups.

#### 3.3.2. Estimated Whole-Body Sweat Sodium and Potassium Concentrations

Based on the estimation approach proposed by Baker et al. [6,19], the calculated whole-body sweat sodium and potassium concentrations of Chinese adults during daily physical activities are summarized in Table 6. The mean Na^+^_whole_ and K^+^_whole_ concentrations for all participants were 34.10 ± 10.31 mmol/L and 3.35 (3.02, 3.93) mmol/L, respectively. Among the three activity groups, the cycling group showed significantly lower Na^+^_whole_ concentrations compared with the running group (*p* < 0.05). The K^+^_whole_ concentrations did not differ significantly among the three activity types (*p* > 0.05).

When stratified by sex (Table 7), male participants exhibited significantly higher Na^+^_whole_ concentrations than females overall after adjustment for body mass (F = 12.849, *p* < 0.001, partial η^2^ = 0.044), whereas estimated whole-body K^+^_whole_ concentrations were lower in males (F = 52.782, *p* < 0.001, partial η^2^ = 0.159).

#### 3.3.3. Distribution of Sodium Concentration Categories

**Figure 3:**
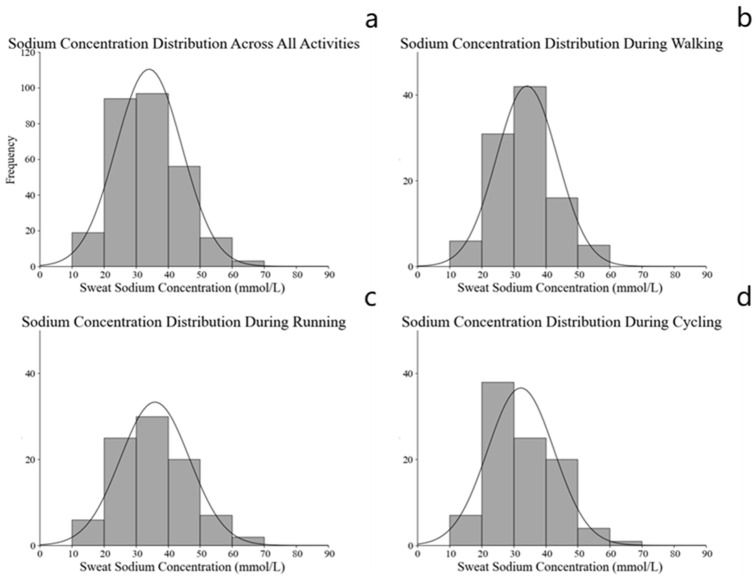
Frequency distribution of estimated whole-body sweat sodium concentrations across all activities (**a**) and by activity type: (**b**) brisk walking, (**c**) running, and (**d**) cycling.

The distribution of estimated whole-body sweat sodium concentrations among Chinese adults during daily physical activities is illustrated in Figure 3. Across all participants, the sweat Na^+^ concentration exhibited a slightly right-skewed distribution, with most values clustering between 20 and 40 mmol/L. The majority of participants fell within the moderate concentration range. The brisk walking and running groups showed a similar pattern, with the greatest number of individuals in the 30–40 mmol/L range, while in the cycling group, most participants exhibited Na^+^ concentrations between 20 and 30 mmol/L.

**Figure 4:**
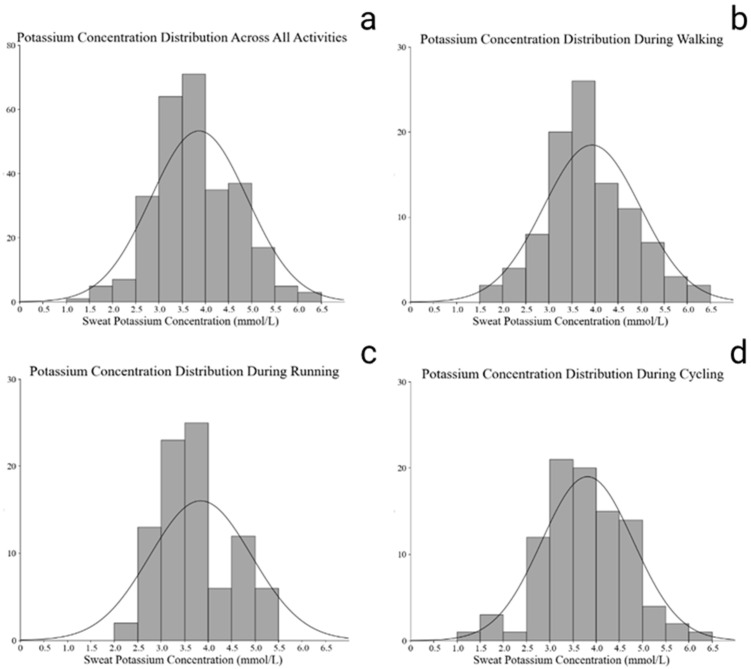
Frequency distribution of estimated whole-body sweat potassium concentrations across all activities (**a**) and by activity type: (**b**) brisk walking, (**c**) running, and (**d**) cycling.

As illustrated in Figure 4, the distribution of estimated whole-body sweat potassium concentrations showed minimal variation across the three exercise types. Unlike sodium, sweat K^+^ values demonstrated a narrow and symmetric distribution, with most participants clustered between 3.0 and 4.0 mmol/L across walking, running, and cycling. This indicates that sweat potassium concentration remained relatively stable regardless of exercise modality.

The categorical distribution of sweat sodium concentrations is summarized in Table 8. Overall, 39.65% of participants were classified as having low Na^+^ concentrations (<30 mmol/L), 59.30% as moderate (30–60 mmol/L), and only 1.05% as high (>60 mmol/L). Among the three activity types, the proportions of moderate sodium concentration were 63.00% in brisk walking, 63.33% in running, and 51.58% in cycling, respectively. In contrast, the proportion of high Na^+^ concentrations remained very low across all activities (<2.5%).

During daily physical activities, most Chinese adults exhibit moderate whole-body sweat sodium concentrations (30–60 mmol/L), with only a small number showing high sodium concentrations, suggesting a relatively consistent sodium excretion pattern across different activity types.

## 4. Discussion

Maintaining optimal hydration status is essential for supporting physiological function, exercise performance, and overall health. Although hydration physiology has been extensively studied in athletes, data on sweat rate and electrolyte composition in non-athletic Chinese adults remain scarce. To our knowledge, this study provides the first population-level dataset describing sweat characteristics during daily physical activities, addressing a key gap in evidence-based hydration research for the Chinese population.

The findings revealed that fluid loss occurred even under mild exercise intensities representative of daily activity. Specifically, 12% of walkers, 72.2% of runners, and 18.9% of cyclists experienced body mass losses exceeding 1%, approaching the threshold at which dehydration begins to impair thermoregulation, cardiovascular stability, and cognitive performance [21,22,23,24,25]. These results highlight that even habitual exercise may cause measurable dehydration, and thus appropriate hydration awareness and scientifically informed fluid replacement practices are important for the general population. The higher sweat rate and BML% observed during running should be interpreted in the context of the higher MET value and greater exercise-related heat production of the running protocol. In the present study, MET values were derived from the CCPA, a Chinese population-specific reference standard based on published evidence and laboratory measurements, including indirect calorimetry. The three activity protocols were standardized using fixed activity-specific speeds to represent common daily physical activity scenarios, but they differed in estimated MET level. Therefore, between-activity differences in sweat responses may reflect both activity modality and differences in activity-specific energy cost or exercise load.

The overall sweat rate among Chinese adults ranged between 0.25 and 1.25 L/h, with most values clustering around 0.5–0.75 L/h, indicating a relatively moderate sweating response. These rates are lower than those reported in tropical or athletic settings—for example, 1.3 ± 0.5 L/h among Thai recreational runners [11] and 1.13 ± 0.58 L/h in athletes [26]. This difference likely reflects the low-moderate intensity of simulated activities, temperature ambient conditions, and the non-athletic background of participants [3,27,28]. The data suggest that real-world daily physical activity produces far lower sweat rates than those used to inform current athletic hydration guidelines, emphasizing the need for population-specific reference values that better reflect daily life conditions.

Clear sex-related differences were observed, with men exhibiting higher sweat rates than women across activity types, and these differences remained evident after adjustment for body mass. However, these findings should not be interpreted as effects of biological sex alone. Previous studies have shown that apparent sex differences in sweating responses may be influenced by body size, body morphology, sweat gland function, and exercise-related metabolic heat production [29,30]. Women, by contrast, possess lower total body water, extracellular fluid volume, and plasma volume, and may experience thermoregulatory strain at smaller absolute fluid deficits [30]. In addition, hormonal factors such as estrogen and progesterone can influence both sweating threshold and sodium reabsorption, leading to lower sweat sodium concentration and reduced sweating rate in women [31]. Therefore, the present findings suggest that sex-associated physiological and anthropometric factors should be considered when interpreting sweat and hydration responses during daily physical activity. However, because this study was observational and did not test hydration strategies or performance outcomes, these results should be viewed as reference data rather than direct evidence for sex-specific hydration recommendations.

In terms of sweat composition, Na^+^ and K^+^ were the predominant electrolytes. The mean Na^+^ concentration was 34.1 ± 10.3 mmol/L and K^+^ between 3 and 4 mmol/L, broadly consistent with prior data from recreational populations [26]. The estimated whole-body sodium concentration (Na^+^_whole_) exhibited a slightly right-skewed distribution, with most individuals within the moderate range (30–60 mmol/L) and fewer than 2% exceeding 60 mmol/L, consistent with findings reported in a Thai population [11]. Because sweat sodium concentration is strongly influenced by sweating rate, exercise intensity, and ambient temperature [26,32,33], the relatively low sodium levels observed in our study are consistent with the low-moderate thermal and metabolic load.

In addition to Na^+^ and K^+^, the present study measured Ca^2+^, Mg^2+^, Fe, Zn^2+^, and Cu^2+^ to provide a more comprehensive profile of sweat mineral composition during daily physical activities. Although these minerals are present in sweat at substantially lower concentrations than Na^+^ and K^+^, previous studies have shown that exercise-induced sweating can contribute to measurable dermal losses of Ca, Mg, Fe, Zn, and Cu, particularly when sweat production is prolonged or repeated over time [4,34,35]. From a physiological perspective, Ca^2+^ and Mg^2+^ are involved in neuromuscular excitability, muscle contraction–relaxation processes, and ATP-dependent enzymatic reactions, whereas Fe, Zn^2+^, and Cu^2+^ are closely related to oxygen transport, oxidative metabolism, antioxidant enzyme systems, and mineral homeostasis [36,37,38]. Therefore, measuring these minerals in sweat provides complementary information on sweat-related mineral loss beyond the major electrolytes. However, these data should be interpreted as local chest sweat mineral concentrations rather than direct indicators of whole-body mineral depletion or systemic mineral status.

The trace mineral composition of sweat followed expected physiological patterns, with concentrations of Ca^2+^, Mg^2+^, Zn^2+^, and Cu^2+^ aligning closely with prior reports, though Fe concentrations were higher than those typically observed in athletes [4]. Baker et al. [34] reported Ca^2+^, Mg^2+^, and Cu^2+^ levels of 88.8 ± 34.9 mg/L, 24.1 ± 10.6 mg/L, and 0.239 ± 0.071 mg/L, respectively, among American athletes—values substantially exceeding those measured in the present study. Such discrepancies likely reflect population-specific physiological and environmental differences, as well as the influence of training adaptation and heat acclimation [39]. The present data therefore provide valuable baseline information on sweat electrolyte profiles in a non-athletic East Asian population performing habitual exercise under temperature conditions.

Beyond these population-level patterns, sex-related differences in sweat electrolyte composition were also evident. Male participants had higher Na^+^ but lower K^+^, Zn^2+^, and Cu^2+^ concentrations compared with females, a pattern differing from earlier reports that observed no sex differences in whole-body Na^+^ concentration [11,26]. This pattern may reflect higher sweat rates in men, whereby ductal secretion rates exceed the capacity for sodium reabsorption [40,41]. Female sex hormones, particularly estrogen, are known to enhance sodium reabsorption through epithelial sodium channel modulation, potentially contributing to lower Na^+^ and higher K^+^ in female sweat [42,43]. These findings emphasize the complex interplay of physiological, endocrine, and environmental factors influencing sweat composition, which should be further explored in future research across different menstrual phases and environmental contexts.

Based on measured sweat rates and electrolyte concentrations, the majority of participants exhibited sweat rates between 0.5 and 0.75 L/h, with sweat Na^+^ and K^+^ concentrations primarily ranging from 20–40 mmol/L and 3–4 mmol/L, respectively. Using these observed values, the estimated electrolyte losses during one hour of daily physical activity were approximately 230–690 mg Na^+^ and 59–117 mg K^+^. While modest, these losses can still contribute to cumulative dehydration if daily fluid intake is insufficient. Given that 72% of Chinese adults fail to meet the recommended daily water intake [44], fluid deficit may accumulate over repeated bouts of activity, especially in warm or humid conditions. According to ACSM guidelines, rapid and complete rehydration for athletes requires fluid replacement equal to approximately 150% of body mass loss after exercise, in order to restore fluid balance efficiently [3]. In the present study, participants engaging in daily physical activities still exhibited measurable sweat losses, indicating that appropriate daily hydration practices remain essential to maintain normal hydration status and overall fluid balance. From a practical perspective, the present findings provide baseline observational data for understanding fluid and electrolyte losses during common daily physical activities under temperate conditions. The relatively low-to-moderate sweat rates, body mass loss, and sodium losses observed in most participants suggest that hydration education for similar daily exercise scenarios should emphasize adequate fluid intake, awareness of individual sweat loss, and consideration of activity type, exercise duration, environmental conditions, and personal sweating characteristics. These findings may also provide preliminary reference information for future studies exploring fluid and electrolyte replacement approaches for daily exercise settings, including whether lower-to-moderate sodium replacement strategies are sufficient for individuals with relatively modest sweat sodium losses.

## 5. Practical Applications

The present findings provide practical reference information for hydration education among Chinese adults engaging in common daily physical activities. The observed sweat rate, body mass loss, and electrolyte profiles may help individuals, exercise instructors, community fitness programs, and workplace wellness initiatives better understand the typical range of fluid and electrolyte losses during brisk walking, cycling, and recreational running under temperate conditions. Rather than supporting a one-size-fits-all approach, these data highlight the importance of considering activity type, exercise duration, environmental conditions, and individual sweating characteristics when promoting adequate fluid intake.

For most participants in the present study, sweat loss and sodium loss were relatively modest, suggesting that hydration education for similar daily exercise scenarios should primarily emphasize timely and adequate fluid intake, with electrolyte replacement considered according to exercise intensity, environmental exposure, and individual sweat losses. In this context, the present data may provide preliminary reference values for future studies examining whether lower-to-moderate sodium replacement approaches are appropriate for daily physical activity settings. 

The observed sex-associated differences in sweat rate and electrolyte profiles further suggest that hydration education should acknowledge interindividual variability rather than relying only on general population averages. These findings may also provide physiological reference data for future validation of wearable sweat-sensing devices designed to monitor sweat loss and support personalized hydration feedback. Future research should incorporate controlled hydration interventions, dietary sodium tracking, objective fitness assessment, menstrual-cycle-based evaluation, and testing across different environmental conditions to develop more individualized hydration guidance for diverse populations.

## 6. Limitations

Several limitations should be considered when interpreting the present findings. This study used a cross-sectional observational design and did not directly evaluate hydration strategies, electrolyte beverage formulations, or exercise performance outcomes. Therefore, the results should be interpreted as baseline reference data on sweat rate and sweat electrolyte profiles during common daily physical activities. In addition, all trials were conducted under temperature and relatively dry indoor conditions (23.0 ± 1.3 °C, 23.6 ± 2.9% relative humidity), which limits the generalizability of the findings to hot, humid, or outdoor environments where sweat rate, body mass loss, thermoregulatory strain, and total electrolyte losses may be substantially greater.

Several methodological factors may also have contributed to individual variability. Participants were allowed to drink water ad libitum during exercise to reflect habitual drinking behavior, but self-selected fluid intake may have influenced body mass loss and sweat rate. Although participants were provided with a standardized meal plan for the day before testing and a standardized breakfast on the experimental day, detailed dietary records, habitual sodium intake, and objective biomarkers of sodium intake were not collected. Sweat samples were collected from the chest using sweat patches, and regional variation in sweat composition should be considered. While published equations were used to estimate whole-body Na^+^ and K^+^ concentrations, these values were estimated, not directly measured. For Ca^2+^, Mg^2+^, Fe, Zn^2+^, and Cu^2+^, no validated site-specific correction equations were available; therefore, these analytes should be interpreted as local chest sweat concentrations. Future studies should include more comprehensive dietary control, habitual sodium intake assessment, objective fitness evaluation, menstrual-cycle tracking, and repeated testing across different seasons, environmental conditions, and exercise intensities.

## 7. Conclusions

This study provides baseline reference data on sweat rate, body mass loss, and sweat electrolyte profiles among healthy Chinese adults performing common daily physical activities under temperate environmental conditions. Overall, participants showed low-to-moderate sweat rates and sodium concentrations, with running eliciting greater fluid loss than brisk walking or cycling and with observable sex-associated differences in sweat rate and electrolyte profiles. These findings support population-specific hydration education for daily exercise settings and provide reference information for future studies on individualized hydration strategies and wearable sweat-sensing technologies.

## Figures and Tables

**Table 1:** Anthropometric and body composition characteristics of participants.

Variable	Brisk Walking (n = 100)	Running (n = 90)	Cycling (n = 95)	Total (N = 285)
Age (year)	24 ± 2	24 ± 2	24 ± 2	24 ± 2
Height (cm)	170.2 ± 9.3	170.4 ± 8.9	171.8 ± 9.5	171.1 ± 7.0
Body mass (kg)	67.31 ± 12.58	66.60 ± 11.90	68.59 ± 13.36	67.51 ± 12.62
BMI (kg/m^2^)	23.12 ± 2.68	22.74 ± 2.59	23.11 ± 2.98	23.00 ± 2.75
Muscle mass (kg)	49.05 ± 11.57	50.08 ± 11.02	51.10 ± 11.82	50.06 ± 11.48
Body fat (%)	23.06 ± 8.06	20.57 ± 7.01	21.13 ± 8.27	21.63 ± 7.86
BSA (m^2^)	1.76 ± 0.21	1.77 ± 0.20	1.79 ± 0.21	1.78 ± 0.21

**Table 2:** BML% and sweat rate among Chinese adults performing daily physical activities.

Variable	Brisk Walking (n = 100)	Running (n = 90)	Cycling (n = 95)	Total (N = 285)
BML%	0.7 ± 0.35 ^a^	1.16 ± 0.36	0.61 ± 0.41 ^a^	0.78 ± 0.45
Sweat rate (L/h)	0.62 ± 0.22 ^a^	0.92 ± 0.29	0.63 ± 0.23 ^a^	0.71 ± 0.28

^a^ Significantly different from the running group (*p* < 0.05).

**Table 3:** Sweat rate (L/h) by sex across different activity types among Chinese adults.

Gender	Brisk Walking (n = 100)	Running (n = 90)	Cycling (n = 95)	Total (N = 285)
Male	0.70 (0.60–0.82) ^a^	1.07 (0.94–1.24)	0.71 (0.60–0.90) ^a^	0.81 (0.66–1.03)
Female	0.50 (0.43–0.55) *^b^	0.65 (0.58–0.83) *	0.47 (0.38–0.55) *^b^	0.52 (0.44–0.63) *

* Significantly different between males and females within the same activity (*p* < 0.05); ^a^ significantly different from the running group within males (*p* < 0.05); ^b^ significantly different from the running group within females (*p* < 0.05).

**Table 4:** Local chest sweat electrolyte concentrations by activity type.

Variable	Brisk Walking (n = 100)	Running (n = 90)	Cycling (n = 95)	Total (N = 285)
**Na^+^_chest_ (mmol/L)**	58.37 ± 18.71	60.72 ± 21.08	54.45 ± 20.39	57.8 ± 20.13
**K^+^_chest_ (mmol/L)**	3.76 (3.29, 4.50)	3.57 (3.27, 4.37)	3.71 (3.20, 4.45)	3.68 (3.25, 4.45)
**Ca^2+^_chest_ (mg/L)**	25.05 (16.95, 37.4)	19.90 (14.00, 39.08)	24.00 (18.01, 41.55)	22.90 (16.70, 38.79)
**Mg^2+^_chest_ (mg/L)**	3.65 (2.33, 6.68)	3.71 (1.87, 7.14)	4.08 (2.64, 6.21)	3.68 (2.39, 6.56)
**Fe_chest_ (mg/L)**	2.28 (0.98, 5.45)	2.03 (1.12, 5.61)	2.53 (1.08, 4.79)	2.27 (1.08, 5.42)
**Zn^2+^_chest_ (mg/L)**	1.72 (1.34, 2.22) ^a^	1.32 (1.04, 1.84)	1.82 (1.33, 2.50) ^a^	1.62 (1.16, 2.14)
**Cu^2^^+^_chest_ (mg/L)**	0.07 (0.04, 0.10)	0.06 (0.04, 0.09)	0.06 (0.04, 0.11)	0.06 (0.04, 0.10)

^a^ Significantly different from the running group (*p* < 0.05); Fe values include both Fe^2+^ and Fe^3+^.

**Table 5:** Sex-specific local chest sweat electrolyte concentrations by activity type.

Variable	Gender	Brisk Walking (n = 100)	Running (n = 90)	Cycling (n = 95)	Total (N = 285)
**Na^+^_chest_ (mmol/L)**	Male	62.87 ± 20.90	66.13 ± 22.41	57.21 ± 22.76	61.83 ± 22.19
	Female	54.05 ± 15.32 *	55.77 ± 18.68 *	51.25 ± 16.94	53.75 ± 16.97 *
**K^+^_chest_ (mmol/L)**	Male	3.45 ± 0.76	3.45 (2.98, 3.77)	3.35 (2.94, 3.93)	3.45 (2.97, 3.84)
	Female	4.41 ± 1.09 *	3.76 (3.44, 4.81) *	4.30 (3.68, 4.66) *	4.16 (3.54, 4.80) *
**Ca^2+^_chest_ (mg/L)**	Male	22.20 (17, 33) ^a^	19.50 (15.15, 38.95)	25.8 (18.55, 41.95) ^a^	22.40 (17.55, 36.3)
	Female	33.90 (16.95, 39.65)	20.20 (13.65, 38.6)	23.85 (17.88, 41.53)	23.85 (15.7, 40.5)
**Mg^2+^_chest_ (mg/L)**	Male	3.23 (2.27, 5.13)	3.53 (1.75, 6.52)	4.08 (2.45, 6.32)	3.43 (2.27, 6.17)
	Female	5.84 (2.46, 7.3) *	3.25 (1.87, 7.14)	4.13 (3.05, 6.06)	4.02 (2.45, 7.04)
**Fe_chest_** **(mg/L)**	Male	2.01 (0.96, 4.55)	2.93 (1.32, 5.2)	2.36 (0.91, 4.45)	2.14 (1.1, 4.98)
	Female	3.96 (1.47, 6.13) *	1.80 (1.02, 6.17)	2.61 (1.45, 5.11)	2.45 (1.19, 5.77)
**Zn^2+^_chest_ (mg/L)**	Male	1.61 (1.28, 1.97) ^a^	1.16 (0.75, 1.45)	1.45 (1.11, 1.87) ^a^	1.42 (1.09, 1.82)
	Female	1.85 (1.35, 2.35) ^b^	1.59 (0.93, 2.10) *	2.15 (1.81, 2.83) *^b^	1.86 (1.33, 2.40) *
**Cu^2+^_chest_ (mg/L)**	Male	0.05 (0.03, 0.07)	0.05 (0.03, 0.08)	0.05 (0.04, 0.09)	0.05 (0.03, 0.09)
	Female	0.08 (0.06, 0.11) *	0.08 (0.05, 0.10) *	0.07 (0.05, 0.12) *	0.08 (0.05, 0.11) *

* Significantly different between males and females within the same activity (*p* < 0.05); ^a^ significantly different from the running group within males (*p* < 0.05); ^b^ significantly different from the running group within females (*p* < 0.05); Fe values include both Fe^2+^ and Fe^3+^.

**Table 6:** Estimated whole-body sweat sodium and potassium concentrations by activity type.

Variable	Brisk Walking (n = 100)	Running (n = 90)	Cycling (n = 95)	Total (N = 285)
**Na^+^_whole_ (mmol/L)**	34.18 ± 9.52	35.94 ± 10.83	32.26 ± 10.40 ^a^	34.10 ± 10.31
**K^+^_whole_ (mmol/L)**	3.41 (3.05, 3.97)	3.26 (3.04, 3.87)	3.37 (2.98, 3.93)	3.35 (3.02, 3.93)

^a^ Significantly different from the running group (*p* < 0.05).

**Table 7:** Sex-specific estimated whole-body sweat sodium and potassium concentrations.

Variable	Gender	Brisk Walking (n = 100)	Running (n = 90)	Cycling (n = 95)	Total (N = 285)
**Na^+^_whole_ (mmol/L)**	Male	36.80 ± 10.46	39.01 ± 11.38	34.02 ± 11.59	36.47 ± 11.26
	Female	31.66 ± 7.83 *	33.12 ± 9.58 *	30.22 ± 8.51	31.7 ± 8.67 *
**K^+^_whole_ (mmol/L)**	Male	3.17 ± 0.58	3.17 (2.81, 3.42)	3.10 (2.79, 3.53)	3.17 (2.80, 3.47)
	Female	3.90 ± 0.83 *	3.41 (3.16, 4.20) *	3.82 (3.34, 4.09) *	3.71 (3.24, 4.20) *

* Significantly different between males and females within the same activity (*p* < 0.05).

**Table 8:** Distribution of estimated whole-body sweat sodium concentration categories.

Variable	Brisk Walking (n = 100)	Running (n = 90)	Cycling (n = 95)	Total (N = 285)
Low Na^+^ (<30 mmol/L)	37.00	34.44	47.37	39.65
Moderate Na^+^ (30–60 mmol/L)	63.00	63.33	51.58	59.30
High Na^+^ (>60 mmol/L)	0.00	2.22	1.05	1.05

## Data Availability

The original contributions presented in this study are included in the article. Further inquiries can be directed to the corresponding author.
